# Involvement of TRPC4 and 5 Channels in Persistent Firing in Hippocampal CA1 Pyramidal Cells

**DOI:** 10.3390/cells9020365

**Published:** 2020-02-05

**Authors:** Alberto Arboit, Antonio Reboreda, Motoharu Yoshida

**Affiliations:** 1 German Center for Neurodegenerative Diseases (DZNE), 39120 Magdeburg, Germany; Alberto.Arboit@dzne.de (A.A.); aprieto@lin-magdeburg.de (A.R.); 2 Otto-von-Guericke University, 39120 Magdeburg, Germany; 3 Faculty of Psychology, Ruhr University Bochum (RUB), Universitätsstraße 150, 44801 Bochum, Germany; 4 Leibniz Institute for Neurobiology (LIN), 39118 Magdeburg, Germany; 5 Center for Behavioral Brain Sciences (CBBS), 39106 Magdeburg, Germany

**Keywords:** TRPC channels, intrinsic persistent activity, cholinergic modulation, patch clamp, hippocampus, TRPC antagonists, working memory

## Abstract

Persistent neural activity has been observed in vivo during working memory tasks, and supports short-term (up to tens of seconds) retention of information. While synaptic and intrinsic cellular mechanisms of persistent firing have been proposed, underlying cellular mechanisms are not yet fully understood. In vitro experiments have shown that individual neurons in the hippocampus and other working memory related areas support persistent firing through intrinsic cellular mechanisms that involve the transient receptor potential canonical (TRPC) channels. Recent behavioral studies demonstrating the involvement of TRPC channels on working memory make the hypothesis that TRPC driven persistent firing supports working memory a very attractive one. However, this view has been challenged by recent findings that persistent firing in vitro is unchanged in TRPC knock out (KO) mice. To assess the involvement of TRPC channels further, we tested novel and highly specific TRPC channel blockers in cholinergically induced persistent firing in mice CA1 pyramidal cells for the first time. The application of the TRPC4 blocker ML204, TRPC5 blocker clemizole hydrochloride, and TRPC4 and 5 blocker Pico145, all significantly inhibited persistent firing. In addition, intracellular application of TRPC4 and TRPC5 antibodies significantly reduced persistent firing. Taken together these results indicate that TRPC4 and 5 channels support persistent firing in CA1 pyramidal neurons. Finally, we discuss possible scenarios causing these controversial observations on the role of TRPC channels in persistent firing.

## 1. Introduction

Persistent firing, a repetitive neural spiking that persists beyond the triggering stimulus, has been observed in vivo during working memory and temporal association tasks in both humans and animals [1,2,3,4,5,6,7,8]. It is believed that persistent firing supports short-term retention of information during these tasks typically for up to tens of seconds [9,10]. Among other areas in the brain, the hippocampus contributes to working memory and temporal association tasks [11,12,13,14,15,16,17], and exhibits persistent firing [3,4,5,6]. Although multiple mechanisms have been proposed, the cellular and molecular mechanisms supporting persistent firing still remain largely unclear (reviewed by [18,19]).

In in vitro experiments, we and others have shown that individual neurons can support persistent firing during cholinergic receptor activation through intrinsic mechanisms within individual neurons in the hippocampus [20,21,22] and in the other areas involved in working memory [23,24,25,26]. Cholinergic receptor activation is crucial for working memory performance and persistent firing in vivo [27,28,29], and it mediates calcium-activated nonspecific cationic current (CAN current; [30,31,32]), while suppressing potassium currents in hippocampal as well as in cortical neurons [33,34,35]. A brief period of spiking activity or membrane depolarization that increases calcium influx causes a further activation of the CAN current, which subsequently triggers so called “plateau” depolarization and persistent firing [32,36,37,38,39]. It has been hypothesized that this intrinsic mechanism of persistent firing may underlie persistent firing in vivo and support working memory [40].

Evidence suggests that the CAN current and persistent firing are supported by the transient receptor potential canonical (TRPC) channels in multiple brain areas (reviewed by [41,42]). The TRPC channels are expressed widely in the brain [43] but in general, TRPC4 and TRPC5 are the predominant subtype in rodent brain [44]. TRPC4 and TRPC5 channels can be activated by G-protein coupled receptors (GPCR) such as the Gq/11 through the phospholipase C (PLC) signaling pathway [45,46,47]. This activation is strongly potentiated by extracellular and intracellular calcium [46,48,49] through a mechanism that increases the open probability of the channel [49] as described for the potentiation of TRPC5 channels by lanthanides [50]. General TRPC channel blockers such as SKF96365, 2-APB, and flufenamic acid have repeatedly been shown to suppress the CAN current [44,51,52] and persistent firing [20,24,25,53]. Using more molecular approaches, it has been demonstrated that transfection of HEK-293 cells with TRPC5 channels mediates, and an overexpression of TRPC5 or TRPC6 enhances, the CAN current. Meanwhile, introducing pore-dead TRPC subunits that function as dominant negative subunit can block the CAN current [52]. In addition, calmodulin dependent translocation of TRPC5 channel mediates the CAN current [54], and the TRPC4/5 PDZ binding peptide (EQVTTRL) inhibits persistent firing [55], indicating the involvement of TRPC4 and 5 channels.

In contrast to these studies, recent studies using TRPC KO mice yields controversial conclusions. While plateau depolarization was suppressed in TRPC4 and TRPC7 KO mice [56,57], Dasari and colleagues (2013) have shown that plateau depolarization was not altered in the medial prefrontal cortex of TRPC1, TRPC5, TRPC6 and TRPC5,6 double KO mice [58]. Egorov and colleagues (2019) have more recently shown that persistent firing in TRPC1,4,5 triple-KO and TRPC1-7 hepta-KO was intact in the entorhinal cortex [59]. Interestingly, however, behavioral studies have shown that TRPC 1,4,5 triple KO mice [60] and TRPC1 KO mice [61] are impaired in working memory tasks, which is very much in line with the idea that TRPC dependent persistent firing supports working memory. Additionally, it has recently been suggested that cholinergically induced persistent firing is supported by the hERG potassium channels [62]. Furthermore, it has been demonstrated that the general blockers of TRPC channels (e.g., SKF96365 and flufenamic acid) used by many studies to claim the involvement of TRPC channels on persistent firing, are not specific and affect other channels [52,63,64]. Therefore, further studies are needed to investigate the involvement of TRPC channels on cholinergically induced intrinsic persistent firing.

In recent years, highly potent and specific TRPC channel blockers became available [65,66]: ML204 [67], clemizole hydrochloride [68] and Pico145 [69], all of which are TRPC4 and 5 blockers with slightly different potencies. However, to our knowledge, effects of these blockers on persistent firing have not been reported. In addition, another potentially useful tool to demonstrate a specific involvement of TRPC in persistent firing would be the TRPC antibodies. Although antibodies have successfully been used to modulate TRPCs [70,71,72], they have not yet been tested on cholinergically triggered persistent firing. Therefore, in this study, we will test the novel highly specific TRPC channel antagonists, and TRPC antibodies to evaluate the involvement of TRPC4 and 5 channels in persistent firing in CA1 pyramidal cells.

## 2. Methods

### 2.1. Animals

Twelve to fourteen-week-old male 129S6/SvEvTac mice (Taconic) were used for the experiments. All experimental protocols were approved by the local ethic committee (Der Tierschutzbeauftragte, Ruhr-Universität Bochum and Deutsches Zentrum für Neurodegenerative Erkrankungen) and by the Ethical Committee on Animal Health and Care of Saxony-Anhalt state, Germany (license number: 42502-2-1388).

### 2.2. Immunohistochemistry

Mice were perfused with 4% paraformaldehyde (PFA) in phosphate buffered solution (PBS, pH 7.4). The brains were post fixed with 4% PFA solution overnight, and transferred to 30% sucrose in PBS (sucrose-solution) for 24 h. Then, 60 µm thick coronal sections were cut with a Leica CM30505 cryostat (Leica Biosystems, Wetzlar, Germany) using Sakura Tissue-Tek OCT Compound and collected as free floating sections in antifreeze-solution (50% glycerol + 50% sucrose-solution). Sections were rinsed (three times in PBS) and quenched by 0.2% H_2_O_2_ in PBS for 30 min (min) at room temperature (RT). Slices were rinsed again (three times in PBS) before getting permeabilized and blocked in 0.3% Triton-X in PBS (PBST) and 3% Bovine serum albumin (BSA) for 30 min. Afterwards primary antibody, anti-TRPC4 (1:500, Almone Labs, Jerusalem, Israel) or anti-TRPC5 (1:1000, Neuromab, Davis, CA, USA), was applied in the same blocking solution overnight at RT. Additional steps were added before the primary antibody application when anti-TRPC5 was used, where the slices were incubated for 30 min with a mouse on mouse blocking reagent (Vector Labs) diluted in PBST. The sections were then rinsed (three times in PBST) before applying the primary antibodies.

After rinsing with PBS, secondary AB was applied in the blocking solution and incubated for 60 min at RT, followed by another rinsing step. Peroxidase-conjugated antibodies were used against the corresponding origin animal of the first AB: Goat Anti-Rabbit (Jackson, 1:1000) or Sheep Anti-Mouse (Jackson, 1:1000), and a signal amplification kit (TSA Plus Cyanide 4 or Cyanide 5, PerkinElmer) was applied. Sections were then incubated in the dark for 7 min, rinsed three times with 0.3% Tween 20 in Tris buffered solution (TBST) and DAPI stained for 20 min. After rinsing with TBST, slices were mounted on glass object slides with glycerol and sealed. All mounted sections were stored at 4 °C until image acquisition.

### 2.3. In Vitro Patch Clamp Recording

#### 2.3.1. Slice Preparation

Mice were deeply anesthetized with Isoflurane and cervical dislocation was performed. The brain was quickly removed from the skull and immersed in oxygenated ice-cold cutting solution containing (in mM) 110 Choline Cl, 7 MgCl_2_ (6H_2_O), 0.5 CaCl_2_, 2.5 KCl, 25 glucose, 1.2 NaH_2_PO_4_, 25 NaHCO_3_, 3 pyruvic acid, 11.5 ascorbic acid, and 100 D-Mannitol. Horizontal brain slices (350 μm thick) were obtained with a vibrating-blade microtome (Leica VT1000S, Leica Biosystems, Wetzlar, Germany). Brain slices were then individually transferred to a holding chamber filled with normal ACSF (nACSF) containing (in mM) 126 NaCl, 1.2 NaH_2_PO_4_, 26 NaHCO_3_, 1.5 MgCl_2_, 1.6 CaCl_2_, 3 KCl, and 10 glucose. In this holding chamber, the slices were kept at 37 °C for 35 min and additionally for at least 30 min at room temperature before being used for recording. The pH of the cutting solution and nACSF were adjusted by a constant saturation with carbogen (95% O_2_/5% CO_2_).

#### 2.3.2. Recording Procedures

Brain slices were transferred to the recording chamber and continuously superfused with nACSF (34.5 ± 1.5 °C). The slice and cells were visualized with an upright microscope (Zeiss Axioskop 2FS plus, Carl Zeiss Microscopy, Jena, Germany) equipped with a 4× objective lens, a 40× water-immersion objective lens, and a monochrome camera (WAT-902H Ultimate). The CA1 region was targeted using the 4× objective lens and the pyramidal layer was identified with the 40× lens. Patch pipettes (3–8 MΩ) obtained from borosilicate glass capillaries (Science Products), were pulled on a P-87 horizontal puller (Sutter instrument). The pipettes were filled with filtered intracellular solution containing (in mM): 120 K-gluconate, 10 HEPES, 0.2 EGTA, 20 KCl, 2 MgCl_2_, 7 PhCreat di(tris), 4 Na_2_ATP, and 0.3 Tris-GTP (pH adjusted to 7.3 with KOH). For labeling purposes, biocytin was added into the intracellular solution at 0.1% concentration. The whole-cell patch configuration was achieved by forming tight seals (>1 GΩ) on the soma and rupturing the membrane with light negative pressure. Liquid junction potential was not corrected. The access resistance was compensated several times during the recordings. Electrical signals were amplified with a Multiclamp 700B amplifier (Axon Instruments, Fremont, CA, USA), low pass filtered at 10 kHz and sampled at 20 kHz, and recorded using WinWCP software (5.6.5-5.13.4, John Dempster, University of Strathclyde, Glasgow, UK). After recording, the slices were stored at 4 °C in a PBS solution with 4% PFA.

#### 2.3.3. Chemicals and Antibodies

All chemicals were purchased from Sigma Aldrich (St. Louis, MO, USA) and Carl Roth (Karlsruhe, Germany) (unless stated otherwise). Carbachol was purchased from Alfa Aesar (Kandel, Germany), Clemizole hydrochloride and ML204 were obtained from Tocris (Wiesbaden-Nordenstadt, Germany), Pico145 was given by Dr. Robin Bon (University of Leeds, Leeds, UK). Carbachol stock solution was prepared in water. Clemizole hydrochloride, ML204 and Pico 145 were diluted in DMSO and, to prevent degradation, they were stored at −20 °C and thawed right before use. DMSO stock solutions were diluted to less than 1:1000 in nACSF.

The antibodies used in the patch clamp experiments were the anti-TRPC4 from Neuromab (USA) and the anti-TRPC5 from Almone Labs (Israel). Both antibodies targeted an epitope in the intracellular part of the channels. The anti-TRPC4 antibodies targeted the C-terminal tail of the TRPC4 channel (Epitope: 930-947), while the anti-TRPC5 antibodies targeted the C-terminal tail of the TRPC5 channel (Epitope: 959-973). Inactivation of the antibodies was carried out by incubating the antibodies at 90 °C for 10 min.

### 2.4. Data Analysis

Data analysis was performed using GNU Octave (4.4.1, John W. Eaton, David Bateman, Søren Hauberg, Rik Wehbring), MATLAB R2014b/R2017b (MathWorks, Natick, MA, USA) (MathWorks), and Prism (7.0, GraphPad Software, San Diego, CA, USA). Cells with spontaneous firing, or resting membrane potential more depolarized than −55 mV, were discarded and not included in the analysis. and Prism (GraphPad Software). pared in water.

Persistent firing was induced using a 100 pA stimulus lasting for 2 s from a membrane potential right below firing threshold (baseline potential). By always bringing the membrane potential just below the firing threshold, persistent firing could be analyzed and compared among different conditions. Persistent firing was quantified using the firing frequency and membrane potential during persistent firing, both of which were measured during a 3 s period after the termination of the stimulation. The frequency of persistent firing was measured as the division of the number of spikes during this 3 s period by 3. The membrane potential depolarization during persistent firing was measured as the difference between the average of membrane potential during this 3 s period, and the average of the baseline potential which was measured in a 3 s period directly before the stimulus onset. Action potentials and fast after hyperpolarization potentials during this 3 s period were not removed from the membrane potential analysis.

Statistical comparisons were performed after inspecting the homogeneity of variance using a Shapiro–Wilk test. Whenever the assumption of homogeneity of variance was met, comparisons were made with parametric tests (unpaired, paired *t*-tests, depending on the data requirements). When the assumption of homogeneity of variance was not met, comparisons were made with non-parametric tests (Wilcoxon test). Significance level α < 0.05 (* *p* < 0.05, ** *p* < 0.01, *** *p* < 0.001) was used. Data is expressed as means ± SEM.

## 3. Results

### 3.1. TRPC4 and TRPC5 Channels Expression in Mouse CA1 Pyramidal Layer

**Figure 1:**
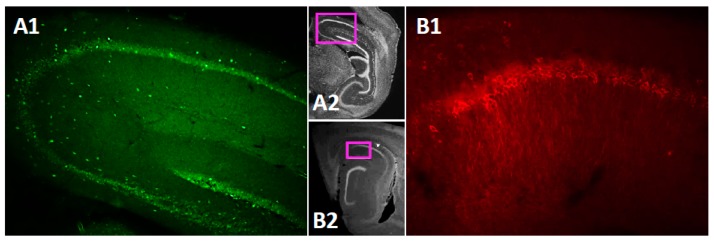
TRPC4 and TRPC5 expression in mouse dorsal hippocampus. (**A1**) TRPC4 expression in a sagittal slice of the hippocampus. (**A2**) Low magnification image indicating the location of the image in A1. (**B1**) TRPC5 expression in CA1 in a sagittal slice of the hippocampus. (**B2**) Low magnification image indicating the location of the image in B1.

The TRPC channels are widely expressed in the brain [43]. However, data on subregion specific expression of the TRPC4 and 5 within the mouse hippocampus is still rather scarce [44,73]. Therefore, before testing persistent firing in neurons in the CA1 pyramidal cells, we performed immunohistochemical (IHC) staining for the TRPC4 and TRPC5 channels in the mouse brain. The IHC staining confirmed that both TRPC4 (Figure 1(A1),(A2)) and TRPC5 (Figure 1(B1),(B2)) are expressed in the CA1 pyramidal cell layer.

### 3.2. Cholinergic Agonist Supports Persistent Firing in Mouse CA1 Pyramidal Neurons

**Figure 2:**
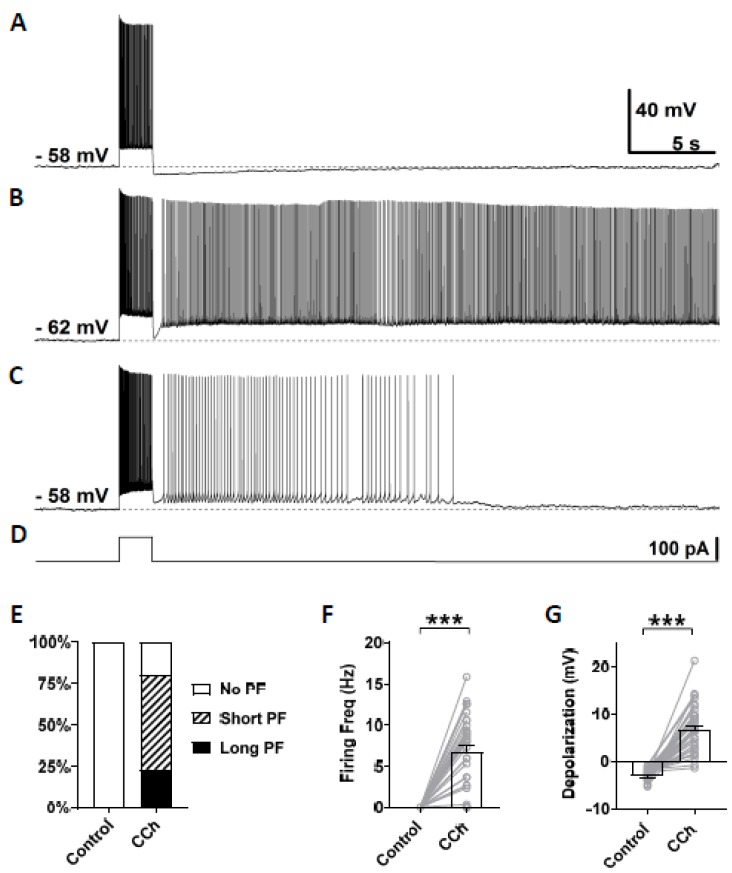
Carbachol dependent persistent firing in mice CA1 pyramidal cells. (**A**) Example of membrane potential response to 2 s depolarization current injection in the control condition (nACSF). Action potentials were fired only during the application of the stimulus, and no persistent firing was observed. (**B**) Example of long-lasting persistent firing during the application of 10 µM carbacol (CCh). (**C**) Example of self-terminating persistent firing in the presence of CCh (10 µM). (**D**) Current injection used in (**A**–**C**). The step depolarization current was 100 pA lasting for 2 s. (**E**) Percentages of cells that showed persistent firing in control condition (nACSF) and during the application of carbachol. (**F**) Post-stimulus firing frequency (Wilcoxon test, *** *p* < 0.001, *n* = 35). (**G**) Post-stimulus depolarization (paired *t*-test, *** *p* < 0.001, *n* = 35). All the recorded cells, shown in (**E**), were included in (**F**,**G**).

In this study, persistent firing was tested in CA1 pyramidal cells in mice brain slices using similar methods to our previous studies conducted in rats [20]. Persistent firing was first tested in the normal artificial cerebrospinal fluid (nACSF). The membrane potential was brought to a level just below the firing threshold using a constant current injection. At this baseline membrane potential, a square current pulse of 100 pA lasting for 2 s was applied to induce persistent firing (Figure 2D). In this control condition, while the current pulse induced a train of action potentials during the stimulation, the membrane potential went back to the baseline after the offset of the current stimulation (Figure 2A, *n* = 35), and none of the tested cells responded with persistent firing (Figure 2E). After the bath application of carbachol (10 µM), 80% of the cells (28/35 cells) responded with persistent firing (Figure 2B,C). In these cells, the membrane potential remained depolarized after the offset of the stimulation, and repetitive action potentials were observed. The remaining 20% of the cells (7/35 cells) did not show persistent firing. Persistent firing was divided into two categories: long-lasting persistent firing which lasted for more than 30 s (Figure 2B), and self-terminating persistent firing which ceased before reaching 30 s (Figure 2C). In mouse CA1 pyramidal cells, 23% of the recorded neurons (8/35 cells) responded with long-lasting persistent firing, and 51% of neurons (20/35 cells) responded with self-terminating persistent firing (Figure 2E). These numbers indicate somewhat lower tendency to exhibit persistent firing in mice compared to rat CA1 neurons where more than 70% of cells responded with long-lasting persistent firing [20]. The firing frequency of persistent firing, measured during the 3 s period after the offset of the stimulation, was 6.76 ± 0.78 Hz (Figure 2F, Wilcoxon, *** *p* < 0.001, *n* = 35). Depolarization during persistent firing was also measured in the same 3 s period. In carbachol, membrane potential depolarization was 6.77 ± 0.88 mV (Figure 2G, paired *t*-test, *** *p* < 0.001, *n* = 35). These results indicate that the majority of CA1 pyramidal cells in mice can support persistent firing during the cholinergic receptor activation as previously shown in rats.

### 3.3. Novel TRPC Antagonists Suppress Persistent Firing

To test the involvement of TRPC4 and 5 channels in persistent firing, we tested recently identified novel TRPC antagonists: ML204 [74], clemizole hydrochloride [74] and Pico145 [68,69,74], which have a higher selectivity than blockers tested previously (e.g., flufenamic acid, SKF-93635, and 2-APB).

**Figure 3:**
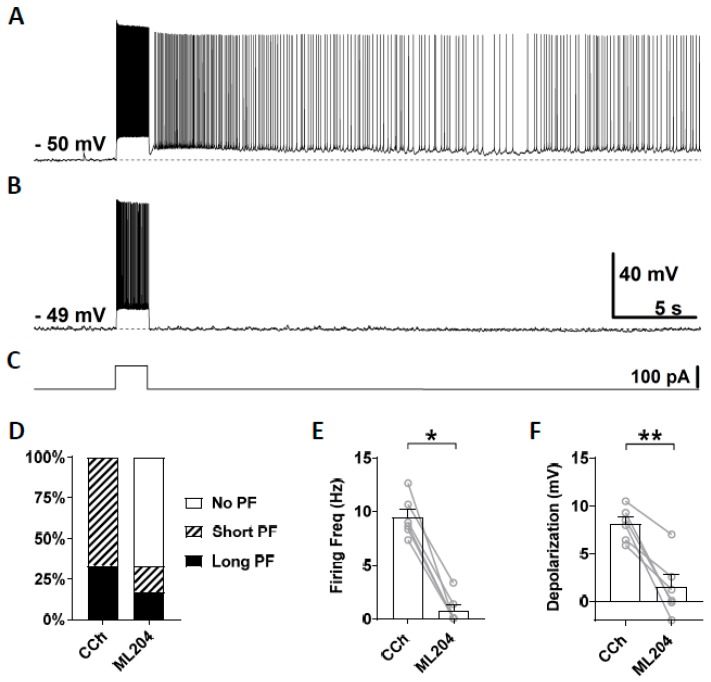
The effect of ML204 on persistent firing. (**A**) Example of persistent firing in CCh (10 µM). (**B**) Suppressed persistent firing in ML204 (10 µM) in the same cell shown in (**A**). (**C**) Current injection used in (**A**,**B**) (100 pA, 2 s). (**D**) Percentages of cells that showed persistent firing in CCh and during the application of ML204. (**E**) Post-stimulus firing frequency (Wilcoxon test, * *p* ≤ 0.05, *n* = 6). (**F**) Post-stimulus depolarization (paired t-test, ** *p* ≤ 0.01, *n* = 6). ML204 significantly suppressed persistent firing.

First, we tested ML204, a blocker of the TRPC4 channel which has a higher selectivity for TRPC4 over TRPC3, TRPC5, and TRPC6 [67,74,75]. At the concentration of 10 μM, ML204 mainly blocks TRPC4 channels. After stable recording was obtained in the normal ACSF, persistent firing was tested in the presence of carbachol (10 μM; Figure 3A). If persistent firing was observed, ML204 (10 μM) was subsequently bath applied in addition to carbachol, and persistent firing was tested 15 min later (Figure 3B). Out of six cells that showed persistent firing, persistent firing was completely blocked in four cells and strongly reduced in the remaining two cells (Figure 3B,D). Both the firing frequency and depolarization, measured during the 3 s period after the stimulation, were significantly reduced by the application of ML204 (Frequency: Figure 3E, Wilcoxon test, *p* ≤ 0.05, *n* = 6; Depolarization: Figure 3F, paired *t*-test, *p* ≤ 0.01, *n* = 6). This effect of the ML204 was reversible in two cells in which we tested a washout of the ML204 (data not shown). These results suggest an involvement of TRPC4 channels in the mechanism underlying persistent firing evoked by cholinergic agonists.

**Figure 4:**
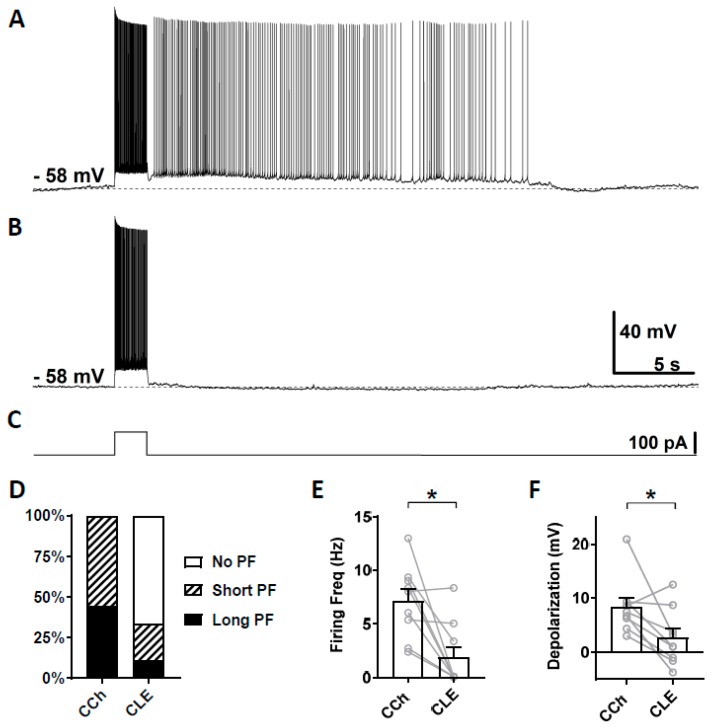
The effect of clemizole hydrochloride on persistent firing. (**A**) Example of persistent firing in CCh (10 µM). (**B**) Suppressed persistent firing in clemizole (CLE, 3 µM) in the same cell shown in (**A**). (**C**) Current injection used in (**A**,**B**) (100 pA, 2 s). (**D**) Percentages of cells that showed persistent firing in CCh and during the application of CLE. (**E**) Post-stimulus firing frequency (Wilcoxon test, * *p* ≤ 0.05, *n* = 9). (**F**) Post-stimulus depolarization (Wilcoxon test, * *p* ≤ 0.05, *n* = 9). Clemizole significantly suppressed persistent firing.

Second, we tested clemizole hydrochloride (CLE), a TRPC5 blocker, which has a higher selectivity for TRPC5 over TRPC4 [68]. The half-maximal inhibitory concentration (IC_50_) for TRPC5 and TRPC4 are 1.1 and 6.4 µM, respectively [68]. When used at 3 µM concentration, clemizole hydrochloride will mainly block TRPC5 channels. As in the section above, persistent firing was first tested in the presence of carbachol (Figure 4A) and then in the presence of clemizole hydrochloride (Figure 4B). In six out of nine cells, persistent firing was completely blocked after 15 min in 3 µM clemizole hydrochloride, while in one out of the nine cells, persistent firing was strongly reduced and, in two remaining cells, persistent firing was not affected (Figure 4D). The firing frequency was strongly and significantly reduced by the application of 3 μM clemizole hydrochloride (Figure 4E, Wilcoxon test, * *p* ≤ 0.05, *n* = 9). The depolarization was strongly reduced by the application of clemizole hydrochloride in all but one cell, which was unaffected (Figure 4F, Wilcoxon test, * *p* ≤ 0.05, *n* = 9). These results suggest an involvement of TRPC5 channels in the mechanism underlying persistent firing.

As described above, both TRPC4 blocker ML204 and TRPC5 blocker clemizole hydrochloride were effective in blocking persistent firing in most of the cells. However, in some cells, persistent firing was not completely blocked or not clearly affected by the blockers. To test whether this remaining activity is due to remaining TRPC4 or 5 channels, we tested dual application of both ML204 and clemizole hydrochloride in these cells. In the two cells which continued to respond with persistent firing after the administration of ML204, persistent activity was completely blocked when clemizole hydrochloride (3 μM) was additionally applied (*n* = 2). In these two cells, the post-stimulus depolarization was also suppressed. In the three cells in which persistent firing was not completely suppressed after the application of clemizole hydrochloride (3 μM), ML204 (10 μM) was additionally applied. Persistent firing was completely blocked, and the post-stimulus depolarization was strongly suppressed in all three cells after the additional application of ML204. Taken together, these data suggest that TRPC4 and TRPC5 support persistent firing in a synergistic manner.

**Figure 5:**
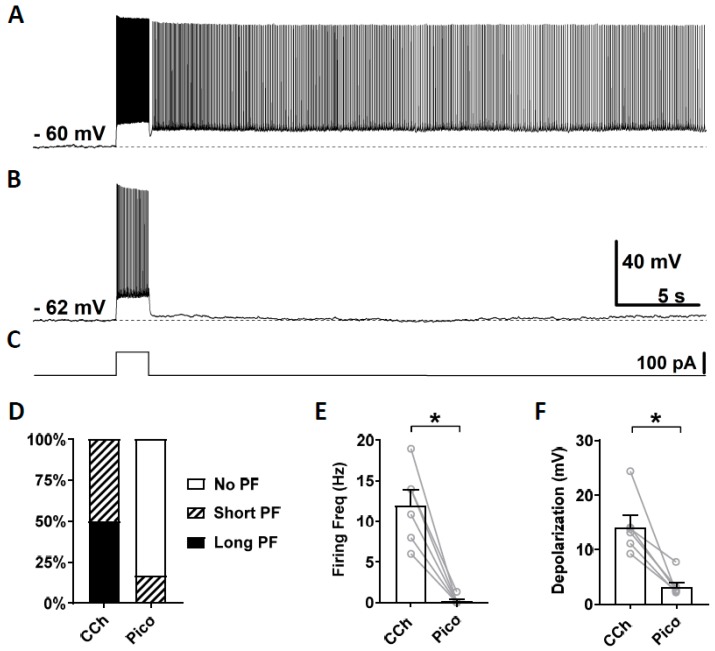
The effect of pico145 on persistent firing. (**A**) Example of persistent firing in CCh (10 µM). (**B**) Suppressed persistent firing in Pico145 (Pico, 100 nM) in the same cell shown in (**A**). (**C**) Current injection used in (**A**,**B**) (100 pA, 2 s). (**D**) Percentages of cells that showed persistent firing in CCh and during the application of Pico. (**E**) Post-stimulus firing frequency (Wilcoxon test, * *p* ≤ 0.05, *n* = 6). (**F**) Post-stimulus depolarization (Wilcoxon test, * *p* ≤ 0.05, *n* = 6). Pico145 significantly suppressed persistent firing.

As the third TRPC channel blocker, we tested a novel TRPC4 and 5 blocker Pico145, which is more potent than ML204 and clemizole hydrochloride. Along with HC-070, Pico 145 is currently the most potent TRPC channel blocker available [69]. Pico145 blocks TRPC channels at nanomolar concentrations while ML204 and clemizole hydrochloride exert their effects at micromolar concentrations. Similarly to the sections above, persistent firing was first tested in the presence of carbachol (Figure 5A) and then in the presence of Pico145 (100 nM; Figure 5B). In five out of six cells, persistent firing was completely blocked after 15–30 min in Pico145, and was strongly reduced in the remaining cell (Figure 5D). The firing frequency and the post-stimulus depolarization were strongly reduced by the application of Pico145 (Figure 5E,F; Firing frequency: Wilcoxon test, *p* ≤ 0.05, *n* = 6; Depolarization: Wilcoxon test, * *p* ≤ 0.05 *n* = 6).

In summary, the strong inhibitory effect of these three novel TRPC channel blockers suggests that persistent firing in the CA1 pyramidal cells is supported by TRPC4 and 5 channels.

### 3.4. Effects of TRPC Antibodies on Persistent Firing

To further assess the involvement of TRPC channels in persistent firing, antibodies for TRPC4 and TRPC5 channels were tested. Anti-TRPC antibodies have successfully been used in patch clamp experiments to selectively reduce the TRPC channel’s activity [70,71,72].

**Figure 6:**
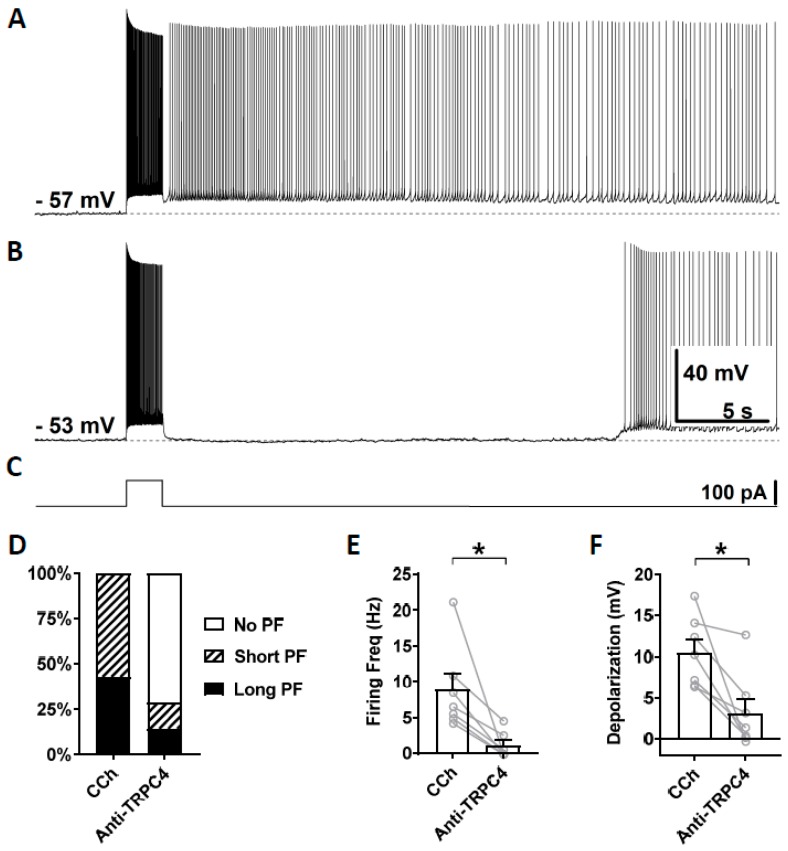
The effect of intracellular application of TRPC4 antibody (1:100) on persistent firing. (**A**) Example of persistent firing recorded 5 min after the rupturing of the membrane. (**B**) Suppressed persistent firing recorded 15 min after the rupturing of the membrane in the same cell shown in (**A**). (**C**) Current injection used in (**A**,**B**) (100 pA, 2 s). (**D**) Percentages of cells that showed persistent firing in control (5 min) and 15 min after the rupturing of the membrane. (**E**) Post-stimulus firing frequency (Wilcoxon test, * *p* ≤ 0.05, *n* = 7). (**F**) Post-stimulus depolarization (Wilcoxon test, * *p* ≤ 0.05, *n* = 7). TRPC4 antibody significantly suppressed persistent firing.

To test the involvement of TRPC4 channels, anti-TRPC4 antibody was diluted (1:100) in the intracellular solution and, after the rupture of the membrane, was able to diffuse inside the cytosol. This dilution, 1:100, was chosen based on a previous work on CA1 pyramidal cells [72]. In this and following experiments with antibodies, carbachol (10 µM) was applied right after the membrane was ruptured and a stable recording was established. Three minutes after the application of carbachol, which was 5 min after the rupturing of the membrane, persistent firing was tested for the first time using the same current injection used in the previous section. This served as the control recording (Figure 6A). Then, 15 min after the rupturing of the membrane, persistent firing was tested again (Figure 6B). Out of the seven cells which showed persistent firing in the control condition, persistent firing was completely blocked in five cells, and the remaining two cells exhibited a weaker persistent firing 15 min after the rupturing of the membrane (Figure 6D). The firing frequency of persistent firing was significantly suppressed compared to the control recordings (Figure 6E; Wilcoxon test, *p* ≤ 0.05, *n* = 7). The post stimulus depolarization was reduced in all seven neurons by the anti-TRPC4 antibody application (Figure 6F, Wilcoxon test, *p* ≤ 0.05, *n* = 7).

**Figure 7:**
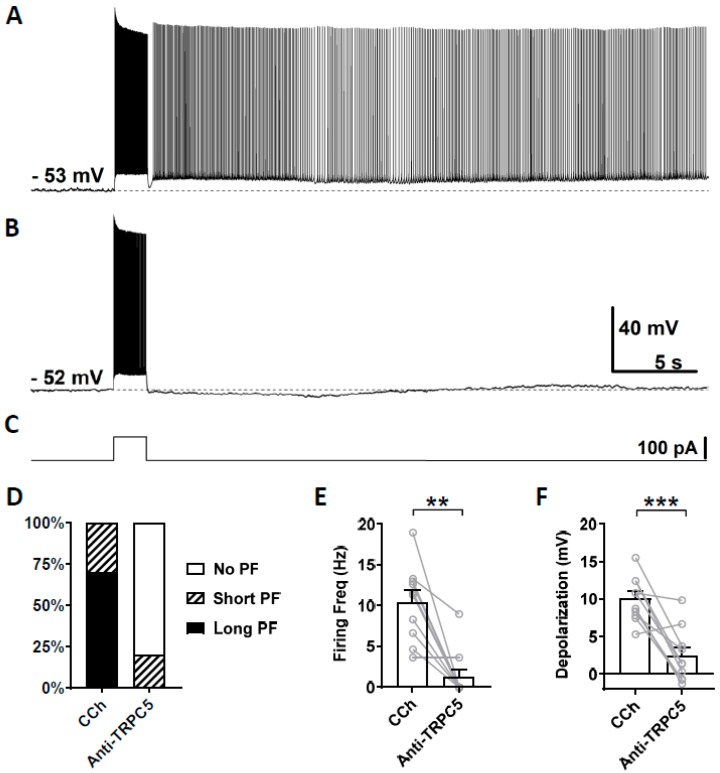
The effect of intracellular application of TRPC5 antibody (1:100–500) on persistent firing. (**A**) Example of persistent firing recorded 5 min after the rupturing of the membrane. (**B**) Suppressed persistent firing recorded 15 min after the rupturing of the membrane in the same cell shown in (**A**). (**C**) Current injection used in (**A**,**B**) (100 pA, 2 s). (**D**) Percentages of cells that showed persistent firing in control (5 min) and 15 min after the rupturing of the membrane. (**E**) Post-stimulus firing frequency (Wilcoxon test, ** *p* ≤ 0.01). (**F**) Post-stimulus depolarization (paired *t*-test, *** *p* ≤ 0.001). TRPC5 antibody significantly suppressed persistent firing.

We then tested the effect of a TRPC5 antibody (1:100–500 dilution) on persistent firing using the same approach. In this experiment, out of 10 cells that responded with persistent firing in the control condition (Figure 7A), persistent firing was completely blocked in eight cells, in one cell it was reduced, while the remaining cells continued to respond with persistent firing (Figure 7B,D). On average, both the firing frequency of persistent firing and the post-stimulus depolarization were significantly reduced (Figure 7E, Firing frequency: Wilcoxon test, *p* ≤ 0.01, *n* = 10; Figure 7F, Depolarization: paired *t*-test, *** *p* ≤ 0.001, *n* = 10).

**Figure 8:**
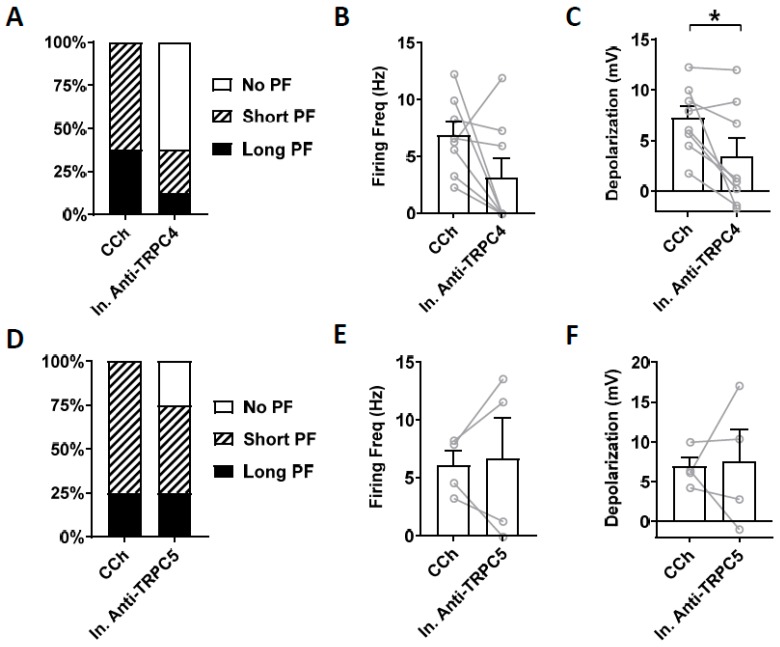
The effect of intracellular application of inactivated antibodies. (**A**–**C**) Inactivated TRPC4 antibody (In. Anti-TRPC4, 1:100). (**A**) Percentages of cells that showed persistent firing in control (5 min) and 15 min after the rupturing of the membrane. (**B**) Post-stimulus firing frequency (Wilcoxon test, ns, *p* = 0.11, *n* = 8). (**C**) Post-stimulus depolarization (paired *t*-test, * *p* ≤ 0.05, *n* = 8). (**D**–**F**) Inactivated TRPC5 antibody (In. Anti-TRPC5, 1:100). (**D**) Percentages of cells that showed persistent firing in control (5 min) and 15 min after the rupturing of the membrane. (**E**) Post-stimulus firing frequency (paired *t*-test, *p* = 0.82, *n* = 4). (**F**) Post-stimulus depolarization (paired *t*-test, *p* = 0.64, *n* = 4).

Heat inactivated antibodies can often, but not always, be used to assess potential non-specific effect of antibodies [72]. Therefore, we tested heat-inactivated TRPC4 and TRPC5 antibodies on persistent firing. Heat-inactivated TRPC4 antibody (1:100 dilution) did not reduce the firing frequency of persistent firing significantly (Figure 8B; paired *t*-test, ns, *p* = 0.11, *n* = 8). The post-stimulus depolarization was, however, reduced significantly (Figure 8C; paired *t*-test, * *p* ≤ 0.05, *n* = 8) although the degree of the reduction was smaller than the antibody without heat treatment (Heat treated: 53 %, Non-heat treated: 70 %). When heat-inactivated TRPC5 antibody (1:100 dilution) was tested, neither the firing frequency nor the post-stimulus depolarization were reduced significantly (Figure 8E; firing frequency: paired *t*-test, *p* = 0.82, *n* = 4; Figure 8F; depolarization: paired *t*-test, *p* = 0.64, *n* = 4).

Taken together, the suppressive effect of the antibodies, and reduced effect of the heat-treated antibodies suggest that persistent firing in CA1 pyramidal neurons are supported by the TRPC4 and TRPC5 channels.

## 4. Discussion

Based on recent controversial reports on the role of TRPC channels in cholinergic dependent persistent firing, we tested three newly identified TRPC4 and 5 antagonists (ML204, Clemizole, and Pico145), and TRPC4 and 5 antibodies, on persistent firing induced in the presence of carbachol in CA1 pyramidal cells. We found that all three TRPC blockers were effective in suppressing persistent firing, with the strongest suppression being observed when both TRPC4 and 5 blockers were co-applied. Intracellular application of TRPC4 and 5 antibodies suppressed persistent firing as well, together indicating that TRPC4 and 5 channels are involved in persistent firing in CA1 pyramidal cells.

### 4.1. Immunohistochemistry

The TRPC channels are widely expressed in the brain [43]. Among the seven TRPC channel subtypes (TRPC1-7), TRPC4 and TRPC5 are highly expressed in the pyramidal layer of the hippocampus [44]. The high expression of TRPC4 and 5 in the hippocampus can also be seen in in situ hybridization images provided by the Allen Brain Atlas (http://portal.brain-map.org/). The immunohistochemistry staining targeting the TRPC4 and TRPC5 channels indicated the expression of both of these channels in the CA1 pyramidal cell layer (Figure 1). These TRPC4 and 5 expression patterns were in general in agreement with previous publications [76].

### 4.2. Persistent Firing in CA1 Pyramidal Cells in Mice

As shown in Figure 2, we observed both long-lasting and self-terminating persistent firing in CA1 pyramidal cells, similar to our previous study in rat CA1 [20]. While the ratio of cells that responded with persistent firing was slightly lower in mice (81%) compared to that in rats (100%), the ratio of cells responding with long-lasting persistent firing in mice (30%) was clearly lower than in rats (73%). In addition, firing frequency and depolarization during the persistent firing in mice (6.8 Hz and 6.8 mV; average measured in cells with persistent firing) were slightly lower than those in rats (10 Hz and 11 mV). This indicates that persistent firing in CA1 pyramidal cells might be somewhat weaker in mice than in rats.

### 4.3. Effects of TRPC4 and 5 Blockers

As mentioned above, commonly used TRPC channels inhibitors, such as SKF-93635, 2-APB, and flufenamic acid, are non-specific, and can affect several channels other than TRPC channels such as the L and T-type calcium channels [52,63,77], voltage-gated sodium channels [78,79], chloride channels [80], cardiac potassium currents [81], and some members of TRPM, TRPV and TRPA channels [64]. In addition, these general blockers do not show selectivity for specific TRPC channel subfamilies. Therefore, we tested more recently identified, and more specific antagonists, ML204, Clemizole, and Pico145, in this study.

ML204 was reported to be a potent and selective TRPC4 channel blocker [67]. As we demonstrated in Figure 3, ML204 (10 µM) strongly inhibited persistent firing and associated depolarization. ML204 exerts its blocking effect by directly interacting with the TRPC4 channels, and the IC_50_ of ML204 for TRPC4 channels is 0.96 µM [67]. At 10 µM, it blocks TRPC4 current completely while blocking TRPC5 current partially (65%; [67]). On the other hand, 10 µM ML204 did not affect TRPV1, TRPV3, TRPA1, TRPM8, KCNQ2, voltage-gated sodium, potassium, and calcium channels in mouse dorsal root ganglion neurons, having a superior selectivity for TRPC channels compared to the classical general blockers mentioned above [67]. Therefore, our data on ML204 provides another piece of evidence supporting the role of TRPC channels in persistent firing.

Clemizole hydrochloride was identified as a TRPC5 blocker [68]. In our study, clemizole (3 µM) significantly reduced persistent firing as shown in Figure 4. Clemizole blocks the TRPC5 channels directly with an IC_50_ of 1.0–1.3 µM, and is six or more times sensitive to TRPC5 over TRPC4 (IC_50_ = 6.4 µM), TRPC3 (IC_50_ = 9.1 µM), TRPC6 (IC_50_ = 11.3 µM), and TRPC7 (IC_50_ = 26.5 µM) [68]. In addition, TRPM3 and M8, and TRPV1, V2, V3, and V4 channels were only weakly suppressed when the concentration of clemizole was increased higher than 20 µM, suggesting that these channels were not suppressed in our study. Therefore, our observations with 3 µM clemizole suggest an involvement of TRPC5 in persistent firing.

Pico145 is the most recently discovered antagonist for TRPC4 and 5 channels, and it is so far the most selective one available, affecting the TRPC channels at pico to nano-molar concentrations [69,82]. Pico145, which is referred to as HC-608 by the inventor, has a very similar potency as HC-070 [82,83,84]. Pico145 is shown to block both the heteromers, TRPC4-TRPC1 heteromer (IC_50_ = 0.033 nM) and TRPC5-TRPC1 heteromer (IC_50_ = 0.199 nM), and the homomers, TRPC4 homomer (IC_50_ = 0.35 nM) and TRPC5 homomer (IC_50_ = 1.3 nM; [69]). The reported IC_50_ values vary slightly among different recording conditions [69] and different groups reported [82]. However, the IC_50_ values for TRPC4 and 5 homomers and heteromers are all below 4.7 nM, indicating that Pico145 is a very potent antagonist. In our study, Pico145 (100 nM) inhibited persistent firing significantly (Figure 5). Pico145 at 100 nM concentration has been shown to have no effect on TRPC3, TRPC6, TRPV1, TRPV4, TRPM2, TRPM8, and TRPA1 [69]. In addition, TRPV3, hERG, Kv1.3, Cav1.2, Nav1.2, Nav1.5 channels are shown to have IC_50_ values that are 11 or more times higher than the concentration we tested [82]. Therefore, our observation with Pico145 provides an additional piece of evidence supporting the involvement of TRPC1, 4, and 5 channels in persistent firing in CA1 pyramidal cells.

### 4.4. Effect of TRPC4 and 5 Antibodies

When antibodies bind to an ion channel, the channel can either be activated or inhibited depending on the targeted epitope. Based on the previous literature, we used antibodies targeting epitopes that led to an inhibition of the ion channel activity [70,71,72]. The suppression of persistent firing by TRPC4 and TRPC5 antibodies we observed (Figure 6 and Figure 7) is in agreement with the previous study in which persistent increase of firing frequency was blocked by TRPC1, 4, and 5 antibodies in CA1 pyramidal cells [72]. The CAN current is known to be induced both under cholinergic and group I mGluR activation through the G_q/11_ family G proteins [85,86,87]. Therefore, while group I mGluR activation was used in the study of El-Hassar and collleagues [72], ionic mechanisms involved in their study might be very similar to the ones involved in persistent firing we studied here.

To demonstrate the specificity of the antibodies, we tested heat-treated antibodies. Heat-treated antibodies were in general less effective in blocking persistent firing. However, while the effect of TRPC5 antibodies was clearly absent after deactivation, heat-treated TRPC4 antibodies still had some effect on the membrane depolarization of persistent firing. While this could potentially mean that the TRPC4 antibody was not as specific, we would like to point out that inactivation of antibody by heat treatment is not always possible. Interestingly, the previous two studies which used the TRPC4 and 5 antibodies from the same provider reported only the results of inactivated TRPC5 antibody [70,72].

### 4.5. Controversies and Limitations

As mentioned above, recent studies in TRPC knockout mice may contradict the role for TRPC discussed above [58,59]. Dasari and colleagues (2013) used TRPC1, TRPC5, TRPC6, and TRPC5 and 6 double KO mice, and demonstrated that plateau potentials measured in the medial prefrontal cortex under a cholinergic activation were intact [58]. This is in contrast with an earlier work, also in the prefrontal cortex, that reported the role of TRPC5 in a plateau potential using dominant negative subunit [52]. Egorov and colleagues (2019) reported that persistent firing measured in the medial entorhinal cortex was unchanged both in TRPC1,4,5 triple-KO, and in TRPC1-7 hepta-KO mice. This contradicts the earlier studies which indicated the role of TRPC4 and 5 channels on persistent firing cortex using pharmacological tools as well as TRPC4/5 binding peptide in the same cell type in the medial entorhinal cortex [24,55]. Nevertheless, the results from the TRPC hepta-KO mice clearly indicate that persistent firing can be supported without any member of the TRPC family at least in the medial entorhinal cortex.

There are at least three explanations for these contradictory observations. The first possibility is that in the medial entorhinal cortex (where persistent firing in the hepta-KO mice was tested), persistent firing does not rely on the TRPC channels, while in other parts of the brain, e.g., hippocampus, it does. The fact that the KO did not reduce or eliminate persistent firing in the medial entorhinal cortex does not mean that persistent firing in all other areas was also unaffected by the lack of TRPC channels. This hypothesis is in line with the observation from other studies with TRPC KO mice in which decreased plateau potential was observed in lateral septal neurons [56,57]. In addition, this is an interesting hypothesis in terms of behavioral studies which report working memory deficits in similar TRPC knockouts [60,61], because suppression of persistent firing in any working-memory related areas could account for the deficit. Since general KO was used in these two studies, it is difficult to test whether persistent firing in all neuron types in all brain areas that are possibly linked to working memory are unchanged or not. Finally, this hypothesis is in agreement with our data presented in this paper.

The second possibility is that persistent firing is supported by the TRPC channels in wild type animals, while other ionic mechanisms support persistent firing in the TRPC KO mice due to some compensatory mechanisms [88]. This hypothesis is in agreement with many previous studies which indicated the involvement of TRPC channels in persistent firing and plateau potential (see Section 1), and with our results. In this scenario, working memory deficit in TRPC KO mice [60,61] may have stemmed from mechanisms other than altered persistent firing, such as the impaired synaptic plasticity in these KO mice [61,89]. However, it may not be easy to explain how TRPC KO can specifically affect working memory without affecting reference memory [60] solely based on the synaptic plasticity deficit. While it remains unclear what alternative mechanism than TRPC could support persistent firing in TRPC KO mice (e.g., TRPC hepta-KO), channels such as TRPM4 and 5 [90], and hERG [62] might be some of the possible mechanisms. It is also interesting to point out that Egorov and colleagues (2019) observed neurons which did not support persistent firing only in the TRPC hepta-KO group [59], where compensation of the mechanisms supporting persistent firing might be more challenging due to the deletion of all TRPC family members, compared to the TRPC1,4,5 triple-KO.

The third possibility is that persistent firing does not rely on TRPC channels in any of the brain areas in both KO and wild type animals, and therefore, studies that indicated the involvement of TRPC channels were all wrong. Although this would contradict our results, it is not possible to exclude this hypothesis. For example, binding assays indicated that ML204 (10 µM) could inhibit G protein-coupled receptors (GPCRs) including α1A and α2A adrenergic receptors, histamine H1 receptors, M2 and M3 muscarinic receptors [67,91]. In addition, clemizole has recently been shown to block hERG channels [92]. Pico145 is more selective and hERG channels are not affected significantly, however, its effects on TRPM4 and 5 channels, for example, have not been reported.

Our data indicates that both TRPC4 and 5 channels contribute to persistent firing in CA1 pyramidal cells, although it is difficult to judge which of these channels contribute to persistent firing more. It may seem that the effect of the TRPC4 blocker ML204 (Figure 3) was slightly stronger than that of TRPC5 blocker Clemizole (Figure 4). However, the effects of TRPC4 and TRPC5 antibodies were very similar to each other (Figure 6 and Figure 7). TRPC4 and TRPC5 can form homotetramers (TRPC4/4, TRPC5/5) [46] or heterotetramers (TRPC4/5) [93]. In addition, TRPC4 and 5 can form heterotetramers together with TRPC1 (TRPC1/4 and TRPC1/5) [94]. The selectivity of the blockers we tested to all of these channel complexes is not fully reported. Therefore, it is difficult to assess whether a specific channel complex has a predominant role in persistent firing.

## 5. Concluding Remarks

Our results using novel TRPC channel blockers and antibodies point to the involvement of the TRPC4 and 5 channels in persistent firing in CA1 pyramidal cells, supporting the first and the second hypotheses mentioned above. However, it does not preclude a role for other ionic mechanisms in persistent firing, especially given potential differences between different regions, cell types, and underlying conditions, such as the activation of neuromodulatory networks [18]. Nevertheless, TRPC channels remain promising and intriguing as a potential mechanism of persistent firing, especially because of recent findings supporting their roles in working memory [60,61]. Future research should focus on resolving the contradictory observations mentioned above and determining the molecular mechanisms supporting persistent firing in individual cells, combining selective pharmacological tools with the TRPC KO technology and other genetic approaches. Furthermore, determining whether TRPC channels are involved in persistent firing in vivo will be of crucial importance.
